# Hierarchical Torque Vectoring Control Strategy of Distributed Driving Electric Vehicles Considering Stability and Economy

**DOI:** 10.3390/s25133933

**Published:** 2025-06-24

**Authors:** Shuiku Liu, Haichuan Zhang, Shu Wang, Xuan Zhao

**Affiliations:** School of Automobile, Chang’an University, Xi’an 710000, China; 2021022011@chd.edu.cn (S.L.); shuwang@chd.edu.cn (S.W.)

**Keywords:** distributed driving electric vehicles, handling stability, economy, torque vectoring control

## Abstract

Coordinating vehicle handling stability and energy consumption remains a key challenge for distributed driving electric vehicles (DDEVs). In this paper, a hierarchical torque vectoring control strategy is proposed to address this issue. First, a tire road friction coefficient (TRFC) estimator based on the fusion of vision and dynamic is developed to accurately and promptly obtain the TRFC in the upper layer. Second, a direct yaw moment control (DYC) strategy based on the adaptive model predictive control (MPC) is designed to ensure vehicle stability in the middle layer, where tire cornering stiffness is updated dynamically based on the estimated TRFC. Then, the lower layer develops the torque vectoring allocation controller, which comprehensively considers handling stability and energy consumption and distributes the driving torques among the wheels. The weight between stability and economy is coordinated according to the stability boundaries derived from an extended phase-plane correlated with the TRFC. Finally, Hardware-in-the-Loop (HIL) simulations are conducted to validate the effectiveness of the proposed strategy. The results demonstrate that compared with the conventional stability torque distribution strategy, the proposed control strategy not only reduces the RMSE of sideslip angle by 44.88% but also decreases the motor power consumption by 24.45% under DLC conditions, which indicates that the proposed method can significantly enhance vehicle handling stability while reducing energy consumption.

## 1. Introduction

With the rapid advancement of distributed driving technology, torque vectoring control has been widely applied in DDEVs as a critical means to enhance vehicle dynamic performance [1,2]. Compared with traditional centralized driving architectures, DDEVs enable independent control of the driving torque at each wheel via individual motors, allowing for the flexible generation of direct yaw moments [3,4]. This significantly improves vehicle handling stability [5,6]. Simultaneously, by optimally distributing the driving torques, the motors can operate within their high-efficiency regions, effectively reducing overall energy consumption and extending driving range. However, under varying driving conditions, how to design a torque vectoring control framework that simultaneously improves handling stability and energy conservation remains a pressing technical challenge for DDEVs.

In recent years, numerous advanced DYC strategies have been developed to enhance vehicle handling stability [7,8]. For instance, a DYC controller based on nonsingular terminal sliding mode control was proposed for four-wheel independently driven autonomous vehicles in [9], where the particle swarm optimization algorithm was utilized to optimize the weights between handling and stability, significantly improving stability performance under extreme conditions. In [10], the DYC controller based on an adaptive sliding mode method was designed for DDEVs, with torque distribution proportional to the vertical load. Considering the challenges posed by tire nonlinearities, a Takagi-Sugeno (T-S) fuzzy robust controller was developed in [11] to compute the direct yaw moment, which improved the handling and stability of the vehicle.

In addition, advanced AI control techniques, such as deep learning, reinforcement learning [12], and safe reinforcement learning [13,14], have been increasingly applied in the field of electric vehicle control. The DYC strategy based on deep reinforcement learning was proposed in [15], and simulation results demonstrated that the strategy significantly enhanced the vehicle’s lateral stability under extreme handling conditions. In [16], a coordinated control strategy combining active front steering and torque vectoring based on LQR was developed, where reinforcement learning was employed to adaptively tune the weighting matrices of the LQR controller. Although these methods effectively enhance the vehicle stability [17], the real-time acquisition of the TRFC remains challenging. Furthermore, variations in road friction conditions directly affect tire nonlinearities, creating additional difficulties for DYC strategies.

To address this, several TRFC estimation methods have been proposed [18,19]. In [20], an estimator based on the Unscented Kalman Filter (UKF) and a tire cornering stiffness estimator based on Back-Propagation Neural Network (BPNN) were developed, coupled with an adaptive MPC controller to maintain yaw stability. Considering chassis control under varying road friction conditions, the integrated TRFC estimation method was proposed in [21]. Other techniques, such as particle filtering [22], extended Kalman filtering, and deep learning, have also been proposed for TRFC estimation. However, traditional dynamic-based estimators often suffer from slow convergence and poor robustness. Given that DDEVs are equipped with intelligent sensors such as stereo cameras and lidar, some studies have applied convolutional neural networks or object detection techniques to classify road surface types [23]. Nevertheless, these methods only provide empirical estimations instead of accurate measurements of TRFC. Consequently, TRFC estimation methods based on the fusion of vision and dynamics are more advantageous for practical applications.

On the other hand, existing torque vectoring control strategies predominantly focus on DYC to improve vehicle handling stability under extreme driving conditions. With increasing demands for energy conservation in electric vehicles, greater emphasis has been placed on optimizing drive torque allocation to enhance motor efficiency. In [24], a hierarchical Nash game-based active front steering and torque vectoring control framework was proposed, where the Nash equilibrium theory determined the optimal steering angle and direct yaw moment at the upper layer, and PSO was employed at the lower layer for optimal torque distribution, aiming to minimize overall energy consumption. In [25], a computationally efficient torque allocation strategy was developed to minimize overall power loss. In [26], a DYC controller focusing on motor efficiency optimization was designed for in-wheel motor-driven electric vehicles. Although these studies effectively reduce energy consumption, coordinating handling stability and energy conservation remains a major challenge in torque vectoring control. On this basis, a torque distribution strategy based on reinforcement learning was proposed in [27], where the reward function simultaneously considers stability and energy. Considering the fact that phase planes effectively reflect vehicle stability states, ref. [28] introduced a stability–economy coordination weight adjustment method based on the vehicle’s sideslip angle and sideslip angular velocity phase plane. Similarly, ref. [29,30] used the sideslip angle-yaw rate phase plane to dynamically assess stability and adjust control priorities between economy and handling stability. Nevertheless, traditional phase–plane-based stability assessments exhibit low accuracy during state transitions, reducing adaptability and control performance. Integrating extensible set theory into the phase plane can significantly enhance stability judgment accuracy, thus improving the handling stability and energy conservation of the vehicle.

To this end, in order to enable a real-time estimate of the TRFC and precisely determine the stable motion status of the vehicle, a hierarchical torque vectoring control strategy is proposed for DDEVs, which can effectively enhance the handling stability and energy conservation. The main contributions are summarized as follows:

1. A TRFC estimator based on the fusion of vision and dynamics is developed to improve convergence speed, estimation accuracy, and robustness of the estimator.
2. The DYC, based on an adaptive MPC, is designed to enhance the handling stability of the vehicle, where tire cornering stiffness is obtained using a hybrid tire model that combines the Magic Formula and the Fiala brush model.
3. Comprehensively considering handling stability and energy conservation, a torque vectoring control strategy based on a quadratic programming algorithm is proposed, and the extended phase plane is established to calculate the weight in economy and stability.

The article has the following parts. Section 2 shows the vehicle dynamic model and the tire model. In Section 3, the hierarchical torque vectoring controller for DDEVs is proposed, which includes the TRFC fusion estimator based on vision and dynamics, the DYC controller based on adaptive MPC, and the torque vectoring controller. The effectiveness of the proposed strategy in this paper is verified through HIL simulation in Section 4, followed by the conclusions in Section 5.

## 2. Vehicle Dynamic Model

**Figure 1:**
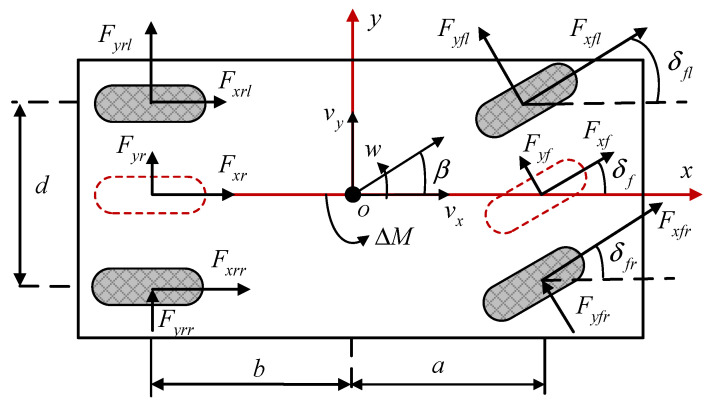
Vehicle dynamics model.

For simplification of the control system, the vehicle dynamics model, as shown in Figure 1, only takes longitudinal, lateral, and yaw motions into account and neglects the body roll, pitch, and vertical motion. The simplified dynamics model of a vehicle can be obtained, which is shown in the following section.

(1) $$\left\{\begin{array}{l} m\left({\dot{v}}_{x}-wv_{y}\right)=\left(F_{xfl}+F_{xfr}\right)\cos\delta_{f}-\left(F_{yf}+F_{yf}\right)\sin\delta_{f}+F_{xrl}+F_{xrr}\\ m\left({\dot{v}}_{y}+wv_{x}\right)=\left(F_{xfl}+F_{xfr}\right)\sin\delta_{f}+\left(F_{yfl}+F_{yfr}\right)\cos\delta_{f}+F_{yrl}+F_{yrr}\\ I_{z}\dot{w}=a\left(F_{xfl}+F_{xfr}\right)\sin\delta_{f}+\frac{1}{2}d\left(F_{xfr}-F_{xfl}\right)\cos\delta_{f}+a\left(F_{yfl}+F_{yfr}\right)\cos\delta_{f}\\ \;\;\;\;+\frac{1}{2}d\left(F_{yfl}-F_{yfr}\right)\sin\delta_{f}-b\left(F_{yrl}+F_{yrr}\right)-\frac{1}{2}d\left(F_{xrl}-F_{xrr}\right) \end{array}\right.$$

where the body coordinate frame $\left(x,y\right)$ is built on the center of gravity (CG). m is the vehicle’s total mass. $v_{x}$ and $v_{y}$ represent the longitudinal and lateral velocity. β and w denote the sideslip angle and yaw rate, respectively. $I_{z}$ is the moment of inertia about the yaw axis. a and b is the distance from the CG to the front and rear axles. d represents the track width of the vehicle. $F_{xi}$ and $F_{yi}$ stand for the longitudinal force and lateral force of the ith wheels, respectively, where, i=fl,fr,rl,rr=1,2,3,4. $F_{yf}$ and $F_{yr}$ denote the generalized lateral force of the front axle and rear axle for simplicity. $\delta_{i}\left(i=fl,fr,f\right)$ is the steering angle of each tire.

**Figure 2:**
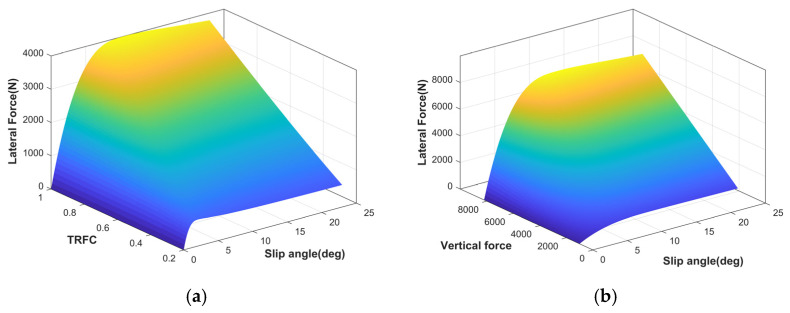
The lateral force of the hybrid tire model. (**a**) TRFC; (**b**) vertical force.

At small slip angles, the lateral force is typically assumed to have a linear relationship with the slip angle. However, the Fiala brush tire model can more accurately capture the nonlinear mechanical characteristics of the tire in its linear region. Therefore, to precisely describe the relationship between lateral force and slip angle, a hybrid tire model combining the Fiala brush model and the Magic Formula is developed [31]. Figure 2 shows the longitudinal characteristics under different TRFC and vertical forces. The specific formulation is as follows:

(2) $$\begin{array}{l} F_{yij}=C_{ij}\left(\mu ,\alpha \right)\alpha_{ij}\\ \left\{\begin{array}{l} -C_{\alpha }\tan\alpha +\frac{{\left(C_{\alpha }\right)}^{2}}{3\mu F_{z}}\left|\tan\alpha \right|\tan\alpha -\frac{{\left(C_{\alpha }\right)}^{3}}{27\mu^{2}{\left(F_{z}\right)}^{2}}{\left(\tan\alpha \right)}^{3},\left|\alpha \leq \alpha_{r,sat}\right|\\ -\mu F_{z}\sin\left[C\arctan\left\{\frac{1}{\mu }B\alpha -E(\frac{1}{\mu }B\alpha -\arctan(\frac{1}{\mu }B\alpha ))\right\}\right]\left|\alpha >\alpha_{r,sat}\right| \end{array}\right. \end{array}$$

where α is tire cornering angle. B,C,E are the fitting coefficients of the Magic Formula. The threshold of the tire slip angle is defined as $\alpha_{r,sat}=\left|\arctan(3mg\mu a/C_{\alpha }\left(a+b\right))\right|$ and $C_{\alpha }=BCF_{z}$.

The tire slip angle and vertical loads on each of the four wheels are given as follows:

(3) $$\left\{\begin{array}{l} \alpha_{f}=\delta_{f}-\left(\beta +\frac{aw}{v_{x}}\right)\\ \alpha_{r}=-\beta +\frac{aw}{v_{x}} \end{array}\right.$$

(4) $$\left\{\begin{array}{l} F_{zfl,fr}=\frac{mgb-mha_{x}}{2\left(a+b\right)}\mp \frac{ma_{y}hb}{\left(a+b\right)d}\\ F_{zrl,rr}=\frac{mga+mha_{x}}{2\left(a+b\right)}\mp \frac{ma_{y}ha}{\left(a+b\right)d} \end{array}\right.$$

where $a_{x}$ and $a_{y}$ denote the longitudinal and lateral accelerations of the vehicle, respectively.

## 3. Hierarchical Torque Vectoring Controller Design for DDEVs

**Figure 3:**
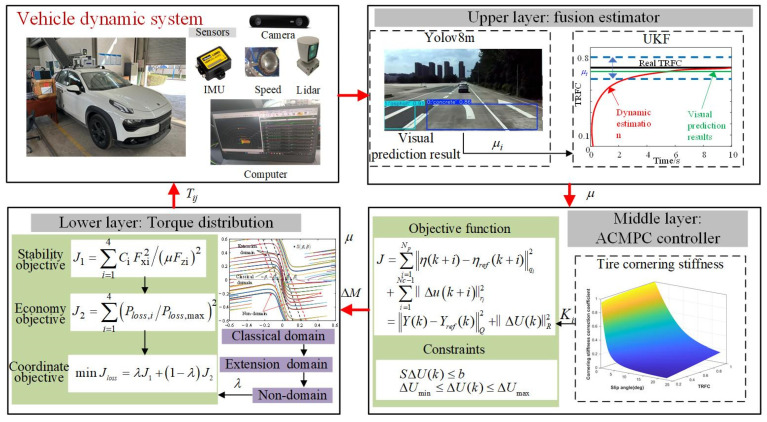
The hierarchical control framework for DDEVs.

The hierarchical control framework for DDEVs, which takes into account handling stability and energy conservation, is illustrated in Figure 3, including three layers.

In the upper layer, the TRFC is estimated based on visual and dynamic information collected by onboard sensors. To accelerate the convergence of the estimator, the road type obtained from vision is used as the initial value for the UKF estimator. The middle layer updates the tire cornering stiffness based on the estimated TRFC and vehicle dynamic states. The direct yaw moment controller based on the adaptive MPC is then established to determine the direct yaw moment, which is subsequently passed to the lower layer. Comprehensively considering the vehicle stability and energy in the lower layer, the torque vectoring controller based on quadratic programming is developed to optimize the distribution of driving torques. The weight between handling stability and energy conservation is dynamically coordinated based on an extended phase-plane, in which the stability boundary is determined by the TRFC. The final optimized torque commands are then applied to the four in-wheel motors of the DDEVs.

### 3.1. Upper Layer: TRFC Fusion Estimator Based on Vision and Dynamics

As a critical factor in vehicle dynamic control, TRFC cannot be directly measured by sensors and must instead be estimated using dedicated estimators or observers. Traditional dynamic algorithms often suffer from slow convergence and poor robustness. In contrast, estimation methods based on vision offer advantages such as long recognition range and high accuracy. Therefore, a TRFC estimator based on the fusion of vision and dynamics is proposed.

#### 3.1.1. Road Type Recognition Method Based on Vision

The vision method can be categorized into object detection and semantic segmentation. Compared to semantic segmentation, object detection offers better real-time performance. Therefore, an object detection method is used for road type classification. Among object detection networks, the YOLO series is widely used due to its fast inference speed.

Compared with YOLOv5 and YOLOv7, YOLOv8m utilizes a Cross Stage Partial (CSP) architecture and PAN-FPN neck network to efficiently extract multiscale semantic information, while reducing redundancy in network parameters to achieve a more compact model [32]. Therefore, the YOLOv8m exhibits a more lightweight network structure, higher detection accuracy, and greater robustness under complex road conditions, and is selected for road type recognition [33].

The training dataset is partially sourced from two public datasets, nuPlan and Kitti, with the majority collected by the experimental vehicle. The dataset includes eight road types: flagging, asphalt, concrete, wet asphalt, wet concrete, wet flagging, snow, and cat ice road. Road type and TRFC range are affected by many factors, and the data [34] are derived through a statistical summary, which is shown in Table 1.

**Figure 4:**
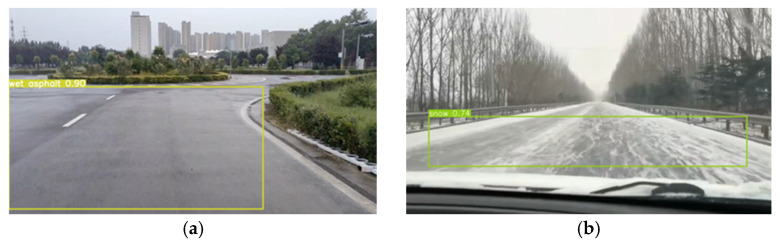
The recognition results of the trained network. (**a**) Wet asphalt road; (**b**) snow road.

During the annotation process, we should align the bounding boxes as closely as possible with the road boundaries, ensuring that the road occupies most of the labeled area while minimizing the inclusion of non-road elements. After annotation, the dataset was randomly divided into a training set and a validation set, with the training set comprising 70% and the validation set 30%. The recognition results of the trained network model are shown in Figure 4, accurately identifying the road types as wet asphalt and snow roads. This enables the determination of the corresponding TRFC ranges.

#### 3.1.2. TRFC Estimation Method Based on UKF

Considering that the UKF can effectively handle the nonlinear propagation of both the mean and covariance, it offers higher estimation accuracy and robustness compared to the conventional Kalman Filter. Therefore, the TRFC estimator based on UKF is developed in this study.

When the road type is continuous, that is, A(k)=A(k−1), the state variable of UKF is defined as follows.

(5) $$x={\left[\mu_{fl},\mu_{fr},\mu_{rl},\mu_{rr}\right]}^{T}$$

When there is a sudden change in road type, that is, A(k)≠A(k−1), the state variable is defined as follows.

(6) $$x={\left[\mu_{sfl},\mu_{sfr},\mu_{srl},\mu_{srr}\right]}^{T}$$

where A(k) denotes the kth recognized road type, and A(k−1) is the $\left(k-1\right)\mathrm{th}$ recognized road type. The initial value of the UKF is defined as $\mu_{fl}$ or $\mu_{sfl}$, that is, the mean friction coefficient corresponding to the current road type. This initialization helps accelerate the convergence of the estimator when the road type changes.

The observation variable is given in Equation (7).

(7) $$z={\left[a_{x},a_{y},\dot{w}\right]}^{T}$$

where $a_{x}$, $a_{y}$, and w are the longitudinal acceleration, lateral acceleration and yaw rate, respectively.

Considering that the road friction coefficient in actual working conditions can be regarded as a slow variable in a short period of time, the system can be expressed as follows.

(8) $$\left\{\begin{array}{l} \dot{x}\left(k\right)=f\left(x,u\right)+w\left(k\right)\\ \dot{z}\left(k\right)=h\left(x,u\right)+v\left(k\right) \end{array}\right.$$

where state equation is $f\left(x,u\right)=I_{4\times 4}x$. $I_{4\times 4}$ is the 4th-order identity matrix. $w\left(k\right)$ is the system process noise with covariance matrix Q and $v\left(k\right)$ is the system measurement noise with covariance matrix R, which can be optimized based on the heuristic optimization algorithm [35]. The measurement equation is defined as follows by Equation (1).

(9) $$h\left(x,u\right)=\left[\begin{array}{cccc} \frac{F_{xfl}\cos\delta_{f}-F_{yfl}\sin\delta_{f}}{m} & \frac{F_{xfr}\cos\delta_{f}-F_{yfr}\sin\delta_{f}}{m} & \frac{F_{xrl}}{m} & \frac{F_{xrr}}{m}\\ \frac{F_{xfl}\sin\delta_{f}-F_{yfl}\cos\delta_{f}}{m} & \frac{F_{xfr}\sin\delta_{f}-F_{yfr}\cos\delta_{f}}{m} & \frac{F_{yrl}}{m} & \frac{F_{yrr}}{m}\\ h(3,1) & h(3,2) & h(3,3) & h(3,4) \end{array}\right]x$$

where $\begin{array}{l} h(3,1)=\frac{a(F_{xfl}\sin\delta_{f}+F_{yfl}\cos\delta_{f})-\frac{d}{2}(F_{xfl}\sin\delta_{f}+F_{yfl}\sin\delta_{f})}{I_{z}}\\ h(3,2)=\frac{a(F_{xfr}\sin\delta_{f}+F_{yfr}\cos\delta_{f})+\frac{d}{2}(F_{xfr}\sin\delta_{f}+F_{yfr}\sin\delta_{f})}{I_{z}}\\ h(3,3)=\frac{-(bF_{yrl}+\frac{d}{2}F_{xrl})}{I_{z}}\\ h(3,4)=\frac{-(bF_{yrr}-\frac{d}{2}F_{xrr})}{I_{z}} \end{array}$.

For this nonlinear system, the detailed computational procedure of the UKF algorithm is described as follows.

First, the sigma points and associated weights are generated through the Unscented Transformation (UT).

(10) $$x_{k}=\{{\hat{x}}_{k},{\hat{x}}_{k}+[\sqrt{(n+\lambda )P_{x,k}}],{\hat{x}}_{k}-[\sqrt{(n+\lambda )P_{x,k}}]\}$$

(11) $$W_{i}^{\left(m\right)}=W_{i}^{\left(c\right)}=\left\{\begin{array}{l} \frac{\lambda }{n+\lambda },i=0\\ \frac{1}{2(n+\lambda )},i=1,\ldots ,2n \end{array}\right.$$

where $P_{x,k}$ denotes the initial state covariance matrix; and $W_{i}^{\left(m\right)}W_{i}^{\left(c\right)}$ represent the weights for the mean and covariance, respectively. λ is a scaling parameter.

Using these sigma points, the one-step state prediction is performed by propagating the points through the process model in Equation (8).

(12) $$x_{i,k}^{-}=f[x_{i,k}^{-}](i=0,\ldots ,2n)$$

where $x_{i,k}^{-}$ denotes the approximate distribution of the sigma points.

Then, the predicted state mean and covariance are computed.

(13) $${\hat{x}}_{k}^{-}=\sum_{i=0}^{2n}W_{i}^{\left(m\right)}x_{i,k}^{-}$$

(14) $$P_{x,k}^{-}=\sum_{i=0}^{2n}W_{i}^{\left(c\right)}[x_{i,k}^{-}-{\hat{x}}_{k}^{-}]{\left[x_{i,k}^{-}-{\hat{x}}_{k}^{-}\right]}^{T}+Q_{k}$$

where ${\hat{x}}_{k}^{-}$ is the a priori state estimate and $P_{x,k}^{-}$ denotes the associated error covariance.

To account for the observation, the predicted state is used to generate a new set of sigma points through UT. These points are then passed through the observation model in Equation (8) to compute the predicted observation’s mean and covariance.

(15) $${\hat{z}}_{k}^{-}=\sum_{i=0}^{2n}W_{i}^{\left(m\right)}H[x_{i,k}^{-}]$$

(16) $$P_{z,k}=\sum_{i=0}^{2n}(W_{i}^{\left(c\right)}\left[H\left(x_{i,k}^{-}\right)-{\hat{z}}_{k}^{-T}\right]*{\left[H\left(x_{i,k}^{-}\right)-{\hat{z}}_{k}^{-T}\right]}^{T})+R_{k}$$

Then, the cross-covariance matrix can be derived as follows.

(17) $$P_{xz,k}=\sum_{i=0}^{2n}W_{i}^{\left(m\right)}[x_{i,k}^{-}-{\hat{x}}_{k}^{-}]{\left[H\left(x_{i,k}^{-}\right)-{\hat{z}}_{k}^{-T}\right]}^{T}$$

where ${\hat{z}}_{k}^{-}$ denotes the predicted measurement and $P_{xz,k}$ is the cross-covariance between the state and observation.

On this basis, the Kalman Gain $K_{k}$ is obtained as follows.

(18) $$K_{k}=P_{xz,k}P_{z,k}^{-1}$$

Finally, the updated state and covariance are obtained by applying the Kalman Gain in Equation (18).

(19) $${\hat{x}}_{k}={\hat{x}}_{k}^{-}+K_{k}[{\hat{z}}_{k}-{\hat{z}}_{k}^{-}]$$

(20) $$P_{x}=P_{x,k}^{-}-K_{k}P_{z,k}K_{k}^{T}$$

where ${\hat{x}}_{k}$ is the a posteriori state estimate, and $P_{x}$ is the updated error covariance.

#### 3.1.3. TRFC Fusion Estimator

**Figure 5:**
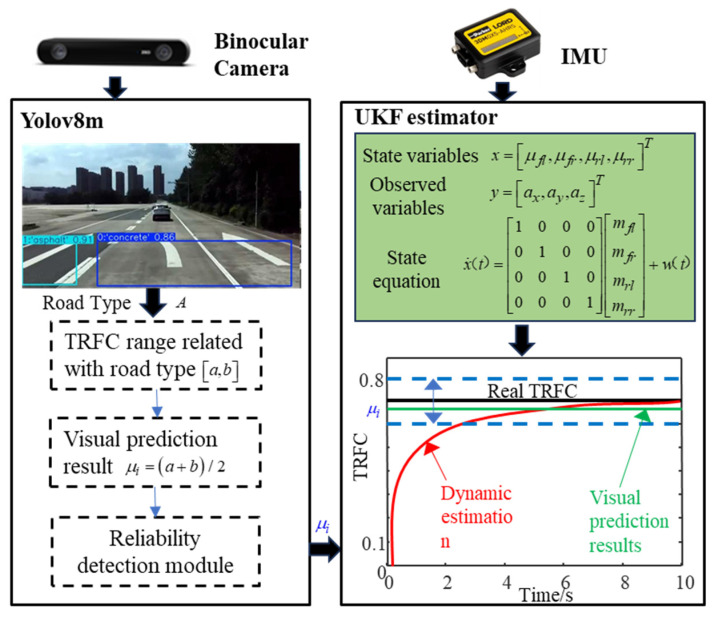
The framework of the TRFC fusion estimator.

**Figure 6:**
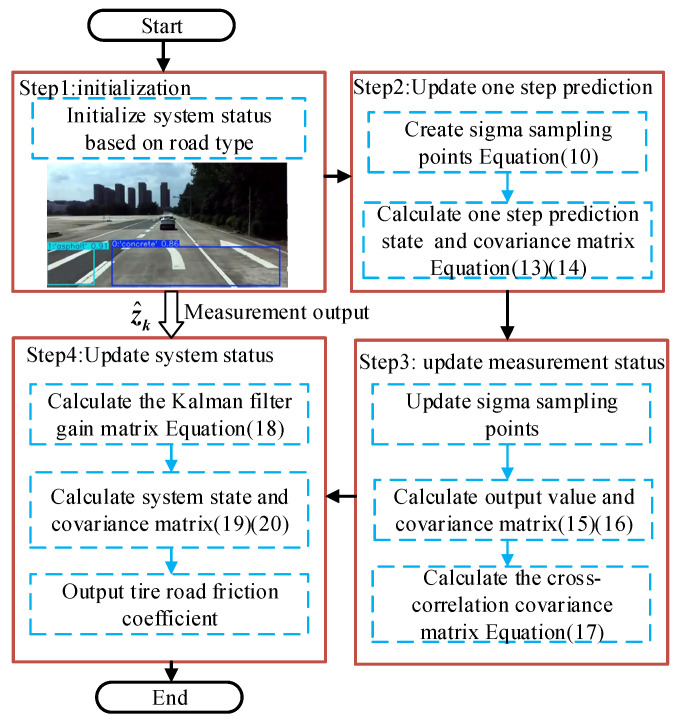
The computational process of the TRFC fusion estimator.

The framework of the TRFC fusion estimator and computational process is illustrated in Figure 5 and Figure 6, respectively. Visual information is fed into the YOLOv8m model to identify the upcoming road type. The corresponding range of TRFC is determined based on the road type. The median value of this range is then used as the initial input for the UKF estimator, enhancing the convergence speed, estimation accuracy, and robustness of the dynamic estimation. In addition, the reliability of the visual output must be verified by setting a confidence threshold. The confidence of the visual detection must satisfy the condition $T_{1}>T_{0},T_{0}=0.4$ before proceeding with the subsequent data fusion.

#### 3.1.4. Fusion Estimator Verification

**Figure 7:**
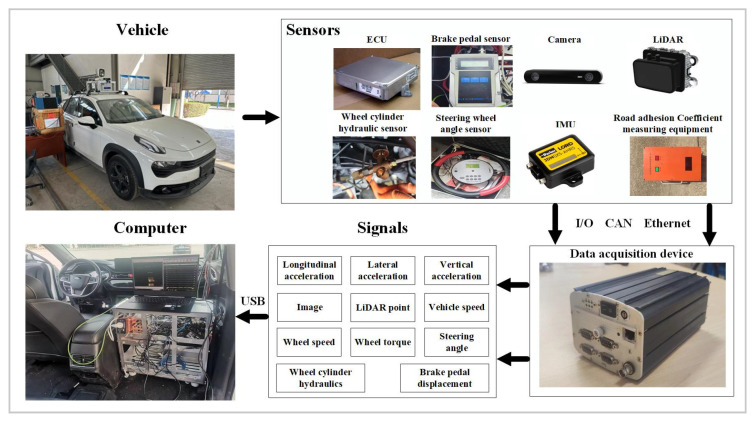
The real vehicle testing platform.

To validate the proposed TRFC fusion estimator, a real vehicle testing platform was constructed, as illustrated in Figure 7. The platform is equipped with multiple sensors, including LIDAR, camera, inertial measurement unit (IMU), RTK real-time kinematic positioning system, and steering angle sensor. The data acquisition device is used to record signals from all sensors. The frequency of the camera and IMU is 30 Hz and 100 Hz, respectively. Detailed hardware specifications are presented in Table 2.

**Figure 8:**
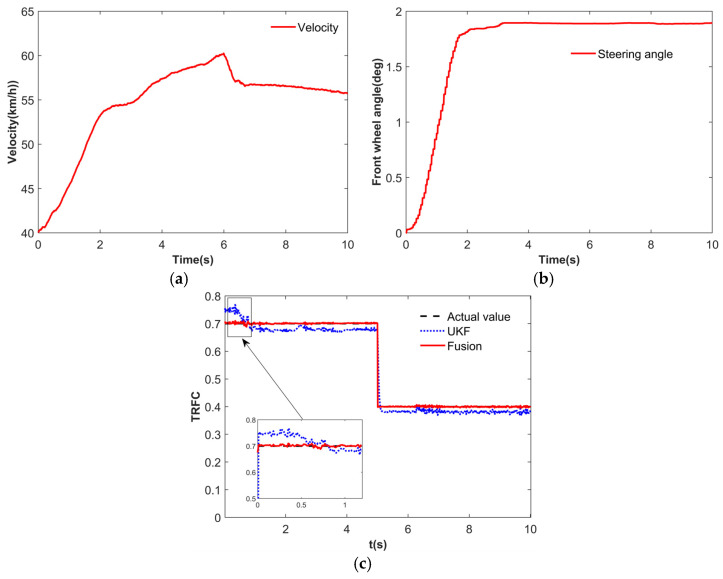
Test results. (**a**) Velocity. (**b**) Steering angle. (**c**) TRFC estimation results.

To emulate real road conditions with abrupt changes in TRFC, the vehicle was tested at the Chang’an University automobile testing ground. The testing track comprised a high-friction section (dry asphalt) and a low-friction section (wet asphalt), which was kept wet by continuously splashing water. To validate the fusion estimator, a step steering maneuver of the front wheels was performed and the test results are presented in Figure 8. Compared to the UKF method, the proposed fusion estimator demonstrated faster convergence and greater robustness. At convergence, the estimation error was 3.5% on the dry road and 3% on the wet road, indicating that the fusion estimator can effectively estimate the TRFC under changing road conditions.

### 3.2. Middle Layer: DYC Controller Based on Adaptive MPC

The direct yaw moment is determined to enhance vehicle handling stability in the middle layer. To meet real-time control requirements, the DYC controller typically employs a linearized tire model to represent tire mechanical characteristics, which compromises control performance when the vehicle operates in nonlinear regions [36].

**Figure 9:**
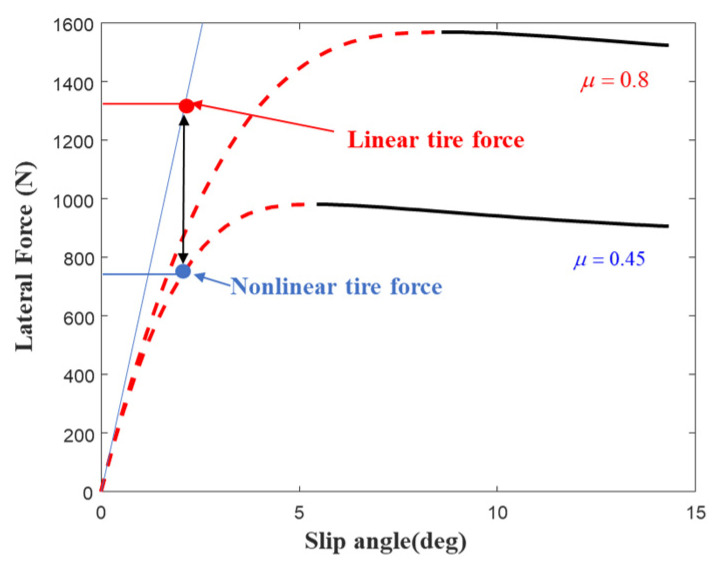
The tire’s nonlinear characteristics under varying TRFC.

Considering that the relationship between tire slip angle and lateral force exhibits different nonlinear characteristics under varying TRFC, using a fixed cornering stiffness to calculate lateral force can lead to significant estimation errors, as illustrated in Figure 9. These errors can further compromise the control performance of the vehicle.

**Figure 10:**
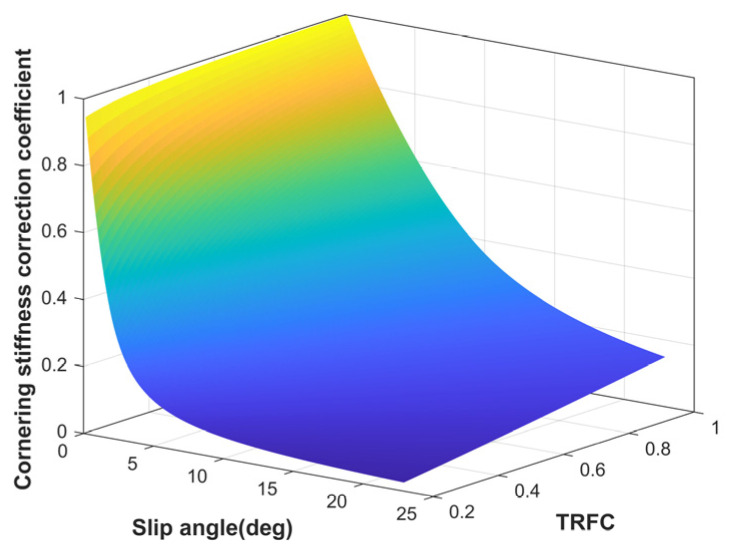
The tire cornering stiffness correction coefficient.

To more accurately capture the tire’s mechanical characteristics in the nonlinear region, a cornering stiffness correction coefficient based on the hybrid tire model is introduced in this paper to calculate the tire lateral force, as illustrated in Figure 10. A lookup table model is employed to dynamically adjust the tire cornering stiffness in real time, which enhances model accuracy and reduces computational load. The actual lateral tire force is defined as follows.

(21) $$\left\{\begin{array}{l} F_{yf}=K_{f}\alpha_{f}=\varepsilon_{f}C_{f}\left(\delta_{f}-\left(\beta +\frac{a\gamma }{v_{x}}\right)\right)\\ F_{yr}=K_{r}\alpha_{r}=\varepsilon_{r}C_{r}\left(-\left(\beta +\frac{a\gamma }{v_{x}}\right)\right) \end{array}\right.$$

where $\varepsilon_{f}$ and $\varepsilon_{r}$ represent the correction coefficients for the front and rear tire cornering stiffness, respectively. $K_{f}$ and $K_{r}$ denote the corrected cornering stiffness of the front and rear tires.

On this basis, the adaptive MPC algorithm, that is Algorithm 1, is proposed to enhance the vehicle handling stability, which is shown as follows.
**Algorithm 1.** Adaptive MPC1:Initialize prediction horizon $N_{p}$ and control horizon $N_{c}$, as well as control weights Q and state weight matrices R.2:Update tire cornering stiffness $K_{f}$, $K_{r}$, vehicle speed $v_{x}$ and the desired reference state $Y_{ref}=\left[w_{ref}^{*},\beta_{ref}^{*}\right]$. Input system state vector $X\left(k\right)$.3:Discrete system (22), and construct the augmented state $\xi \left(k\right)$ in Equation (24).4:Calculate the system outputs in prediction horizon by Equation (26).5:Solve the optimization problem (27) and calculate the optimal control sequence $\Delta U{\left(k\right)}^{*}$.6:Apply the first element of the sequence $\Delta U_{k}^{*}$ to the system (22).7:k=k+1 return to step 2.

A simplified two-degree-of-freedom (2-DOF) vehicle dynamics model, which captures lateral and yaw motions, is established in the middle layer, which can be expressed as follows.

(22) $$\left\{\begin{array}{l} \dot{X}=AX+BU+ED\\ Y=CX_{\prime } \end{array}\right.$$

where $\left\{\begin{array}{l} X={\left[\begin{array}{cc} \beta & w \end{array}\right]}^{T};U=\left[\Delta M\right];D=\left[\delta_{f}\right];Y={\left[\beta ,w\right]}^{T};C=diag(1,1)\\ A=\left[\begin{array}{cc} -\frac{K_{f}+K_{r}}{mv_{x}} & \frac{bK_{r}-aK_{f}}{mv_{x}^{2}}-1\\ a_{21}=\frac{bK_{r}-aK_{f}}{I_{z}} & \frac{-\left(a^{2}K_{f}+b^{2}K_{r}\right)}{I_{z}v_{x}} \end{array}\right];B=\left[\begin{array}{l} 0\\ \frac{1}{I_{z}} \end{array}\right];E=\left[\begin{array}{l} \frac{K_{f}}{mv_{x}}\\ \frac{aK_{f}}{I_{z}} \end{array}\right] \end{array}\right.$.

To reduce the complexity in the continuous system, Equation (22) is discretized as follows:

(23) $$\left\{\begin{array}{l} \dot{X}(k+1)=A_{k}X(k)+B_{k}U(k)+E_{k}D_{k}\\ Y\left(k\right)=CX\left(k\right) \end{array}\right.$$

where $A_{k}=e^{A\Delta T}$, $B_{k}=E\int_{k\Delta T}^{\left(k+1\right)\Delta T}e^{A\left[\left(k+1\right)\Delta T-t\right]}dt$, and ΔT denotes the sampling time.

To facilitate the optimization of the objective function, the state variables and control variables in Equation (23) are combined, resulting in the following new state equation.

(24) $$\left\{\begin{array}{l} \xi \left(k+1\right)={\hat{A}}_{k}\xi \left(k\right)+{\hat{B}}_{k}\Delta u\left(k\right)+{\hat{E}}_{k}D(k)\\ \eta \left(k\right)=C_{k}\xi \left(k\right) \end{array}\right.$$

where the variables n and m denote the dimensions of the state vector and control input, respectively.

(25) $$\left\{\begin{array}{l} \xi \left(k\right)=\left(\begin{array}{l} x\left(k\right)\\ u\left(k-1\right) \end{array}\right),{\hat{A}}_{k}=\left(\begin{array}{cc} A_{k} & B_{k}\\ 0_{m\times n} & I_{m} \end{array}\right),{\hat{B}}_{k}=\left(\begin{array}{l} B_{k}\\ I_{m} \end{array}\right)\\ \Delta u\left(k\right)=u\left(k\right)-u\left(k-1\right),C_{k}={\left[C\;\;\;\;0_{(n+m)\times 1}\right]}^{T} \end{array}\right.$$

At this point, the control output over the entire prediction horizon can be expressed as follows.

(26) $$Y(k)=\psi_{k}\xi \left(k\right)+\Theta_{k}\Delta U\left(k\right)+\Gamma_{k}D(k)$$

where $N_{p}=5$ and $N_{c}=2$ represent the prediction horizon and the control horizon, respectively.

$$Y(k)=\left[\begin{array}{c} \eta (k+1)\\ \eta (k+2)\\ \eta (k+3)\\ \vdots \\ \eta (k+N_{p}) \end{array}\right],\Delta U\left(k\right)=\left[\begin{array}{c} \Delta u\left(k\right)\\ \Delta u\left(k+1\right)\\ \Delta u\left(k+2\right)\\ \vdots \\ \Delta u\left(k+N_{c}-1\right) \end{array}\right],\psi (k)=\left[\begin{array}{c} C_{k}{\hat{A}}_{k}\\ C_{k}{\hat{A}}_{k}^{2}\\ C_{k}{\hat{A}}_{k}^{3}\\ \vdots \\ C_{k}{\hat{A}}_{k}^{N_{p}} \end{array}\;\right]$$

$$\Theta_{k}=\left[\begin{array}{ccc} C_{k}{\hat{B}}_{k} & 0 & 0\\ C_{k}{\hat{A}}_{K}{\hat{B}}_{k} & \ldots & 0\\ \vdots & \vdots & \vdots \\ C_{k}{\hat{A}}_{K}^{N_{P}-1}{\hat{B}}_{k} & \ldots & C_{k}{\hat{B}}_{k} \end{array}\right],\Gamma_{k}=\left[\begin{array}{ccc} C_{k}{\hat{E}}_{k} & 0 & 0\\ C_{k}{\hat{A}}_{K}{\hat{E}}_{k} & \ldots & 0\\ \vdots & \vdots & \vdots \\ C_{k}{\hat{A}}_{K}^{N_{P}-1}{\hat{E}}_{k} & \ldots & C_{k}{\hat{E}}_{k} \end{array}\right]$$

The objective of the middle layer is to minimize control energy consumption while eliminating the deviation between the actual vehicle state and the desired reference state. Therefore, the following cost function is formulated.

(27) $$\begin{array}{l} \underset{\Delta U\left(k\right)}{\min}J={\left\|Y(k)-Y_{ref}(k)\right\|}_{Q}^{2}+{\left\|\Delta U(k)\right\|}_{R}^{2}\\ \;\;\;\;\;\;=\frac{1}{2}{\left(\begin{array}{c} \Delta U\left(k\right)\\ \varepsilon \end{array}\right)}^{T}H\left(\begin{array}{c} \Delta U\left(k\right)\\ \varepsilon \end{array}\right)+f\left(\begin{array}{c} \Delta U\left(k\right)\\ \varepsilon \end{array}\right)\\ s.t.\left\{\begin{array}{l} S\Delta U(k)\leq b\\ \Delta U_{\min}\leq \Delta U(k)\leq \Delta U_{\max\;} \end{array}\right. \end{array}$$

where the desired reference state $Y_{ref}=\left[w_{ref}^{*},\beta_{ref}^{*}\right]$, $Q=diag\left[10,15\right],R=diag\left[500,5000,8000\right]$.

$$\left\{\begin{array}{l} H=\Theta_{k}^{T}Q\Theta_{k}+R\\ f=2\left(Y\left(k\right)-Y_{ref}\left(k\right)-\Theta_{k}\Delta U\left(k\right)\right)Q\Theta_{k} \end{array}\right.,\;\left\{S=\left[\begin{array}{l} A\\ -A \end{array}\right];b=\left[\begin{array}{l} U_{\max}-U_{t}\\ -U_{\min}+U_{t} \end{array}\right]\right.;$$

$$A={\left[\begin{array}{cccc} I & 0 & \ldots & 0\\ I & I & \ldots & 0\\ \vdots & \vdots & \ddots & \vdots \\ I & I & \ldots & I \end{array}\right]}_{N_{c}\times N_{c}};U_{\min}={\left[\begin{array}{l} u_{\min}\left(k\right)\\ u_{\min}\left(k\right)\\ \vdots \\ u_{\min}\left(k\right) \end{array}\right]}_{N_{c}};U_{\max}={\left[\begin{array}{c} u_{\max}\left(k\right)\\ u_{\max}\left(k\right)\\ \vdots \\ u_{\max}\left(k\right) \end{array}\right]}_{N_{c}};U_{t}={\left[\begin{array}{l} u\left(k-1\right)\\ u\left(k-1\right)\\ \vdots \\ u\left(k-1\right) \end{array}\right]}_{N_{c}}$$

Subject to the constraints of the TRFC, the desired yaw rate and sideslip angle are defined as follows.

(28) $$\left\{\begin{array}{l} w_{ref}^{*}=\min\left\{\frac{v_{x}\delta_{f}}{\left(1+Kv_{x}^{2}\right)l},\;\frac{0.85\mu g}{v_{x}}\right\}\\ \beta_{ref}^{*}=\min\left\{\frac{b+mav_{x}^{2}/\left(K_{r}l\right)}{l\left(1+Kv_{x}^{2}\right)}\delta_{f},\;\left(\frac{b}{v_{x}^{2}}+\frac{ma}{K_{r}l}\right)\mu g\right\} \end{array}\right.$$

At each sampling instant, Equation (27) is solved using a receding horizon optimization approach to obtain a sequence of direct yaw moment increments $\Delta U{\left(k\right)}^{*}={\left(\Delta U_{k}^{*},\Delta U_{k+1}^{*},\cdots ,\Delta U_{k+N_{c}-1}^{*}\right)}^{T}$. The first element of this sequence is applied to the controlled plant, and the optimization process is repeated in the next cycle, thereby achieving continuous control of the vehicle’s handling stability.

### 3.3. Lower Layer: Torque Vectoring Controller Considering Stability and Economy

#### 3.3.1. Objective of Stability and Economy

The driving torques among the four wheels are allocated to track the total longitudinal driving force and direct yaw moment in the lower layer. At the same time, it must account for physical constraints such as road adhesion conditions and the maximum torque limits of the motors. To achieve the desired vehicle motion control objectives, tire utilization is often adopted as a control target to enhance vehicle stability. However, the economic conservation of the drive motors, an important optimization metric, is frequently overlooked in practical studies [37]. To address this, an integrated optimization objective that considers vehicle handling stability and energy conservation is established.

Considering that the TRFC utilization can effectively reflect the tire’s safety margin, the stability objective function is defined as follows, where the lateral force can be neglected [38].

(29) $$J_{1}=\sum_{i=1}^{4}C_{i}\frac{T_{i}^{2}}{r^{2}{\left(\mu F_{\mathrm{zi}}\right)}^{2}}$$

where r is the wheel radius. $C_{i}$ represents the weighting coefficient for each wheel. The left and right wheels on the same axle share the same weight, while the rear axle is assigned a higher weight than the front axle to enhance vehicle stability. In this study, the front axle weighting coefficient is set to 1, while the rear axle coefficient is adaptively selected within the range of 1–2 based on the deviation from the phase-plane stability boundary.

**Figure 11:**
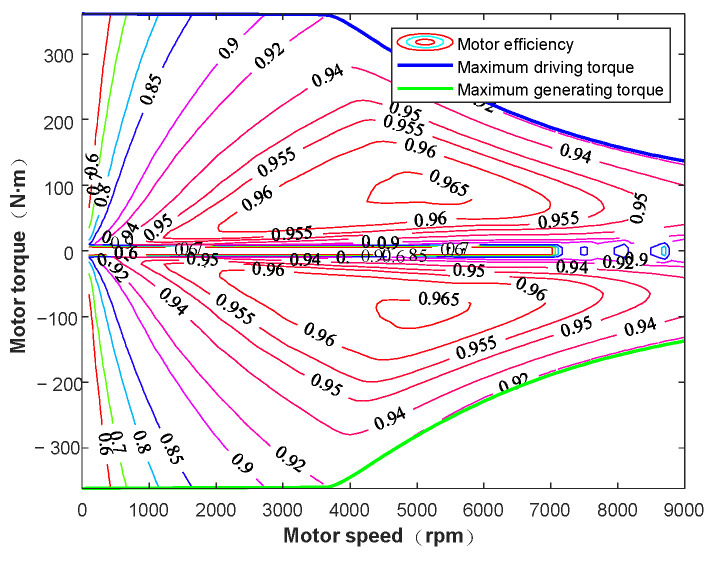
The motor efficiency map.

The motors installed in DDEVs are mainly involved in two power flows. One is the positive power used to drive the vehicle forward, and the other is the negative power used to recover braking energy. In both cases, the motor experiences energy losses due to iron losses, copper losses, and mechanical losses. The actual motor efficiency is defined as follows.

(30) $$\left\{\begin{array}{l} \eta \left(T_{i+},w_{i}\right)=\frac{T_{i+}w_{i}}{T_{i+}w_{i}+P_{cu}+P_{fe}+P_{mech}}\\ \eta \left(T_{i-},w_{i}\right)=\frac{T_{i-}w_{i}+P_{cu}+P_{fe}+P_{mech}}{T_{i-}w_{i}} \end{array}\right.$$

where $P_{cu}$, $P_{fe}$, and $P_{mech}$ denote the iron losses, copper losses, and mechanical losses, respectively. $\eta \left(T_{i\pm },w_{i}\right)$ is the motor efficiency, which depends on torque and speed, and can be obtained from the efficiency map shown in Figure 11. For simplicity, motor response delays are neglected here.

The power losses of the motor under both driving and regenerative braking conditions can be derived as follows.

(31) $$P_{loss,i}=P_{cu}+P_{fe}+P_{mech}=\left\{\begin{array}{l} T_{i+}w_{i}\left(\frac{1}{\eta \left(T_{i+},w_{i}\right)}-1\right),T_{i+}>0\\ T_{i-}w_{i}\left(\eta \left(T_{i-},w_{i}\right)-1\right),T_{i+}<0 \end{array}\right.$$

Therefore, to minimize the power loss of the motors, the following objective function related to energy conservation is defined as follows.

(32) $$J_{2}=\sum_{i=1}^{4}{\left(\frac{P_{loss,i}}{P_{loss,\max}}\right)}^{2}$$

where $P_{loss,\max}$ represents the maximum power loss and is used to normalize the objective function.

On this basis, to balance the performance indicators of stability and energy, the weighted objective function is then defined as follows.

(33) $$J_{loss}=\lambda J_{1}+\left(1-\lambda \right)J_{2}$$

where λ represents the weighting coefficient.

The constraints must be satisfied, which not only considers the dynamic constraints of longitudinal velocity and virtual yaw moment control, but also the physical constraints of motor performance and road adhesion limit. The dynamic constraints of the control system are denoted as follows.

(34) $$\left\{\begin{array}{l} \left(T_{1}+T_{2}\right)\cos\delta_{f}+\left(T_{3}+T_{4}\right)=T_{\exp}\\ \frac{d}{2}\left(-\frac{T_{1}}{r}+\frac{T_{2}}{r}\right)\cos\delta_{f}+\frac{d}{2}\left(-\frac{T_{3}}{r}+\frac{T_{4}}{r}\right)=\Delta M \end{array}\right.$$

The physical constraints of the control system are denoted as follows.

(35) $$\left|T_{i}\right|\leq \min\left(\mu_{i}F_{zi}r,\;T_{i_{-}\max}\right)$$

where $T_{\mathrm{all}}$ denotes the total driving torque. $T_{\max}$ represents the maximum torque of the motor.

On this basis, the optimal wheel torque distribution can be efficiently obtained through a quadratic programming method.

#### 3.3.2. Determination of Weighting Coefficient Based on the Extended Phase-Plane

**Figure 12:**
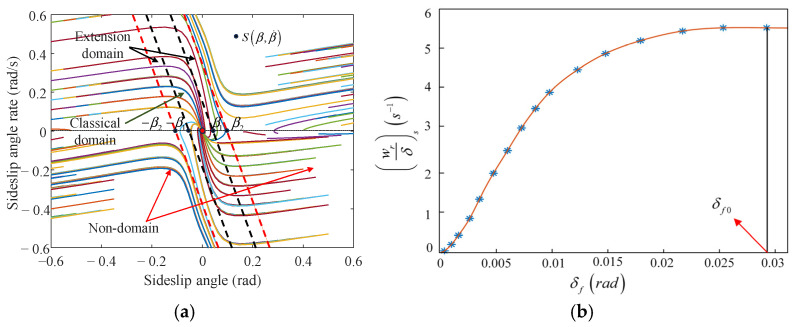
The extensible phase plane. (**a**) The $\beta -\dot{\beta }$ phase plane. (**b**) The relationship between the front wheel steering angle and yaw rate gain.

The weighting coefficient between stability and economy is primarily related to the vehicle’s stability status. When the vehicle operates within the stable region, a smaller λ is assigned to prioritize energy conservation. Conversely, during instability, a higher weight λ is applied to enhance vehicle stability. Traditional phase plane methods used for evaluating vehicle stability often suffer from limited adaptability and low estimation accuracy. To address these shortcomings, this section introduces a stability judgment method based on an extensible phase plane. As shown in Figure 12a, the $\beta -\dot{\beta }$ phase plane is divided into the classical domain, extension domain, and non-domain using a dual-line approach.

In Figure 12a, the boundaries of the classical domain and the extension domain are primarily defined by $X=[-\beta_{1},\beta_{1}]$, which is determined by the tire’s cornering characteristics. The relationship between the front wheel steering angle and yaw rate gain is obtained based on Carsim, as shown in Figure 12b. Based on the steady-state front wheel angle $\delta_{f0}$, the steady-state sideslip angle $\beta_{1}$ can be derived from the linear 2DOF model. On the other hand, the boundary between the extension domain and the non-domain is defined as $X_{0}=[-\beta_{2},\beta_{2}]$, which is determined by the stability limits of the phase plane. In this study, a dual-line method is used to divide the stability region of the phase plane. The equations for these boundary lines are given as follows.

(36) $$|\dot{\beta }+B_{1}\beta |⩽B_{2}$$

where the boundary coefficients $B_{1}$ and $B_{2}$, which are determined by the TRFC, are shown in Table 3.

A characteristic variable ψ(s) is defined to represent the vehicle’s handling stability, thereby enabling the simplification of the two-dimensional domain into a one-dimensional domain. The expression is given as follows.

(37) $$\psi (s)=\beta +B_{1}\dot{\beta }$$

where ψ(s) is the feature quality.

Based on the extension theory, the positional relationship between a point and an interval can be described using the concept of extension distance. The extension distance from any characteristic variable to the classical domain $X=[-\beta_{1},\beta_{1}]$, and to the positive domain $X_{0}=[-\beta_{2},\beta_{2}]$, is defined as follows.

(38) $$\left\{\begin{array}{l} \rho (\psi ,X)=\left|\psi \right|-\beta_{1}\\ \rho (\psi ,X_{0})=\left|\psi \right|-\beta_{2} \end{array}\right.$$

where ρ is the extension distance.

In order to indicate the degree of association between the vehicle’s motion state and the extension planes set, the correlation function can be derived by Equations (37) and (38).

(39) $$K(\psi )=\frac{\rho (\psi ,X_{0})}{D(\psi ,X,X_{0})}$$

where $D(\psi ,X,X_{0})=\rho (\psi ,X_{0})-\rho (\psi ,X)$.

The correlation function K(ψ) reflects the degree of association between the system’s current state and the sets defined in the extensible phase plane, which accounts for the transition process of the vehicle’s lateral stability status and categorizes the vehicle’s state into three types: stable region: K(ψ)≥1, transitional region: 0<K(ψ)<1, and unstable region: K(ψ)≤0. Therefore, the weighting coefficient can be obtained.

(40) $$\lambda =\left\{\begin{array}{cc} 1 & K(\psi )\leq 0\\ \lambda =1-K(\psi ) & 0<K(\psi )<1\\ 0 & K(\psi )\geq 1 \end{array}\right.$$

## 4. Simulation Results and Analysis

**Figure 13:**
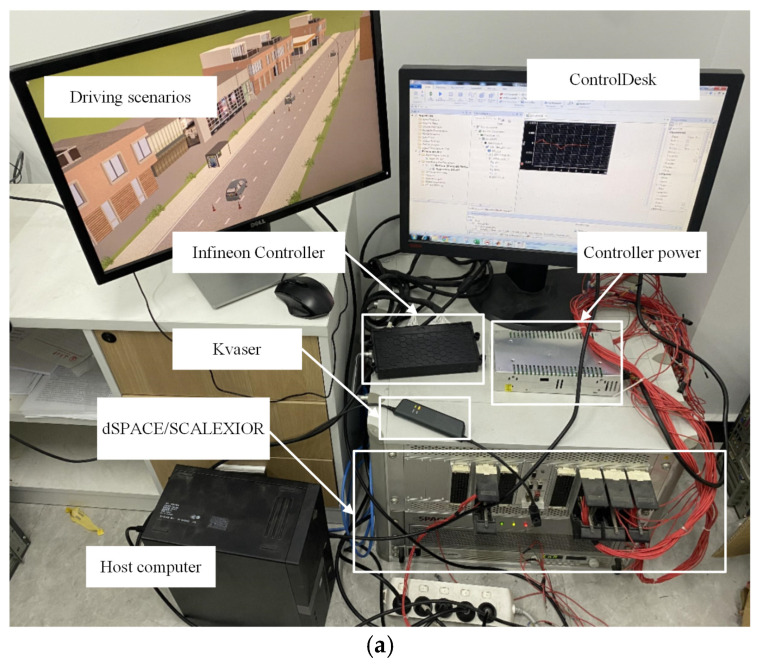

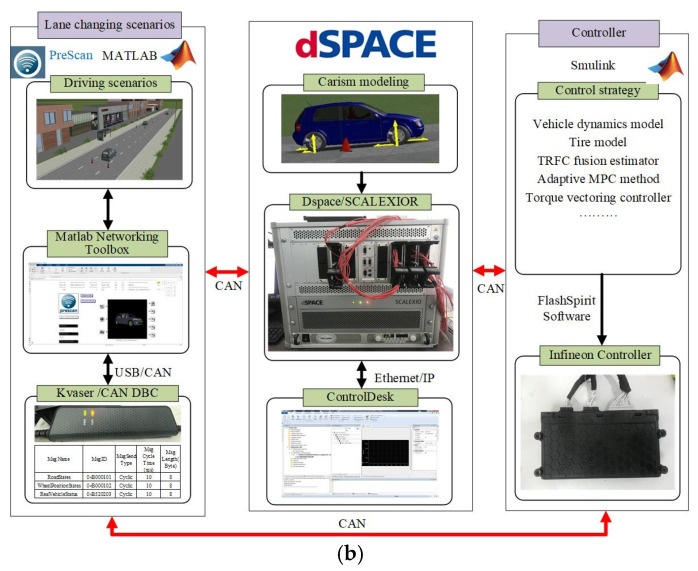
HIL simulation experiment. (**a**) HIL test platform. (**b**) Implementation process of HIL simulation system.

To verify the effectiveness of the proposed control strategy, a Hardware-in-the-Loop (HIL) test platform was established, as shown in Figure 13a. The platform consists of dSPACE hardware, software systems, and vehicle control hardware. The detailed implementation process is illustrated in Figure 13b. In this setup, a nonlinear vehicle dynamics model is configured in CarSim 2019 and deployed on the dSPACE platform. The software system is built using the Prescan/Simulink (version number 2407) co-simulation environment, which is used to create lane-changing scenarios. Communication between the simulation platform and the dSPACE system is achieved through Kvaser Leaf Light V2, with a baud rate set to 500 kb/s. The hardware system includes an Infineon vehicle controller. The proposed control strategy is implemented in MATLAB/Simulink (R2024a version number 24.1) and converted into C code via the Real-Time Workshop (RTW), with a sampling time set to 10 ms. It is worth noting that ControlDesk (2024-B version number 24.2) is used to monitor and manage the entire HIL simulation process. The vehicle parameters and key controller parameters are given in Table 4.

To evaluate the economic conservation of the proposed torque distribution strategy, the FTP (Federal Test Procedure) driving cycle is adopted for energy testing. Additionally, a Double Lane Change (DLC) scenario is established to further verify the effectiveness of the proposed control strategy. The torque vectoring controller, simultaneously considering handling stability and energy conservation, is defined as Energy-Stability Allocation (ESA).

To highlight the advantages of the proposed ESA strategy, a baseline strategy focusing solely on handling performance, defined as Stability Allocation (SA), is conducted for comparisons. In the SA strategy, the conventional MPC is used in the middle layer, whereas the ESA strategy employs an adaptive MPC controller proposed in this paper.

### 4.1. FTP Driving Cycle

**Figure 14:**
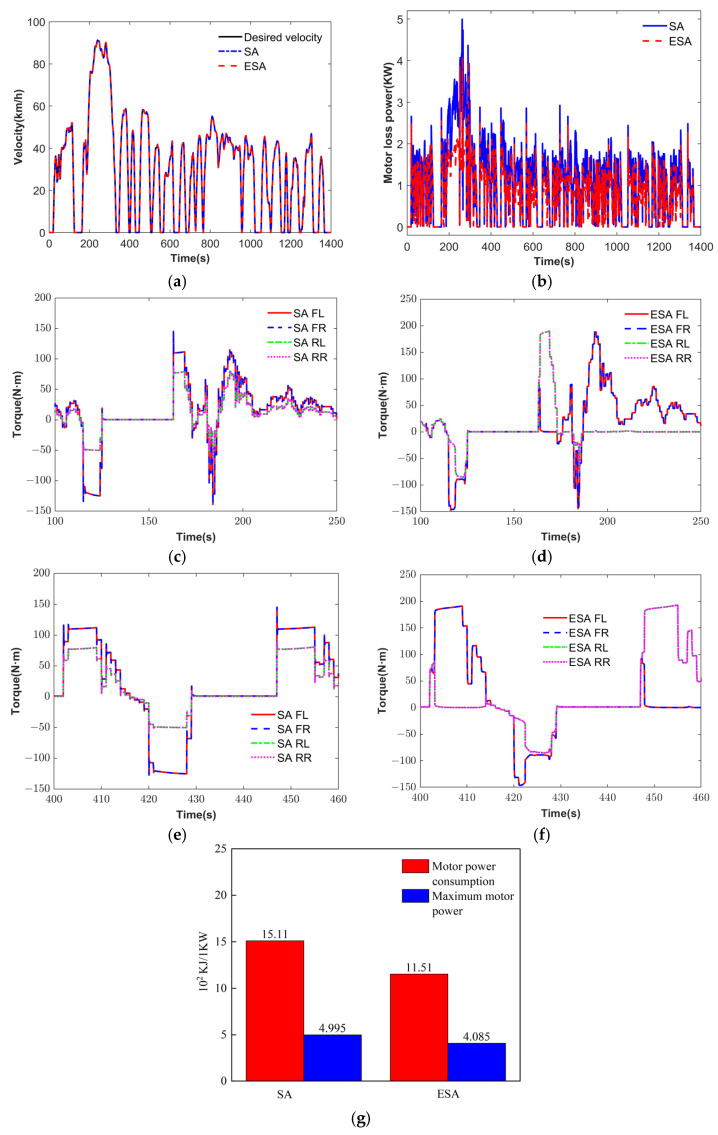
FTP simulation results. (**a**) Velocity. (**b**) Motor loses power. (**c**) SA torque at 100 s. (**d**) ESA torque at 100 s. (**e**) SA torque at 100 s. (**f**) ESA torque at 400 s. (**g**) Economic performance indices.

To verify the effectiveness of the proposed torque vectoring controller, the FTP driving cycle is implemented as a test scenario. Since only longitudinal control is involved and the vehicle remains within the stable region throughout the cycle, the weighting coefficient λ between handling and energy is set to 1. The simulation results under the FTP cycle are presented in Figure 14.

As shown in Figure 14a, both the EA and ESA strategies can closely track the target velocity across the entire driving cycle, meeting the longitudinal performance requirements. Figure 14b illustrates the motor power loss under the two strategies. As illustrated in Figure 14g, compared to the stability-oriented torque distribution (SA) strategy, the torque vectoring controller based on ESA achieves a reduction of 18.22% in peak motor power loss and 23.83% in motor power consumption, which highlights the superior energy conservation of the proposed torque vectoring controller. Figure 14c–f shows the local driving torque distribution results. During acceleration and braking phases, the ESA strategy allocates higher driving torques than the EA strategy, indicating that the motors operate in more efficient regions. This contributes to a significant improvement in the overall energy conservation of the vehicle.

### 4.2. DLC Simulation

**Figure 15:**
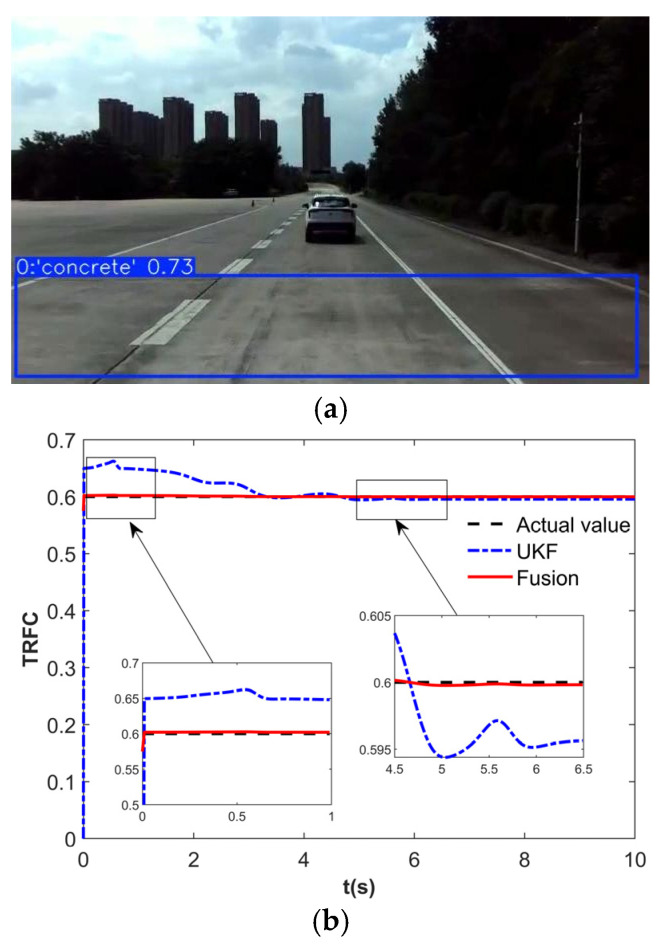
TRFC estimation results. (**a**) Vision estimation. (**b**) Fusion estimation.

To validate the effectiveness of the proposed control strategy, the DLC simulation was conducted. The TRFC estimation results are shown in Figure 15. When the road is identified as concrete based on vision, the initial TRFC for the UKF estimator is set to 0.575, as shown in Figure 15a. As illustrated in Figure 15b, during the time interval of 0–1 s, the fusion estimator exhibits a faster convergence to the true TRFC compared to the UKF estimator. At around 4.5 s, the fusion estimator demonstrates a smaller steady-state error. These results indicate that the fusion of vision and dynamics significantly enhances the convergence speed and estimation accuracy of the TRFC.

**Figure 16:**
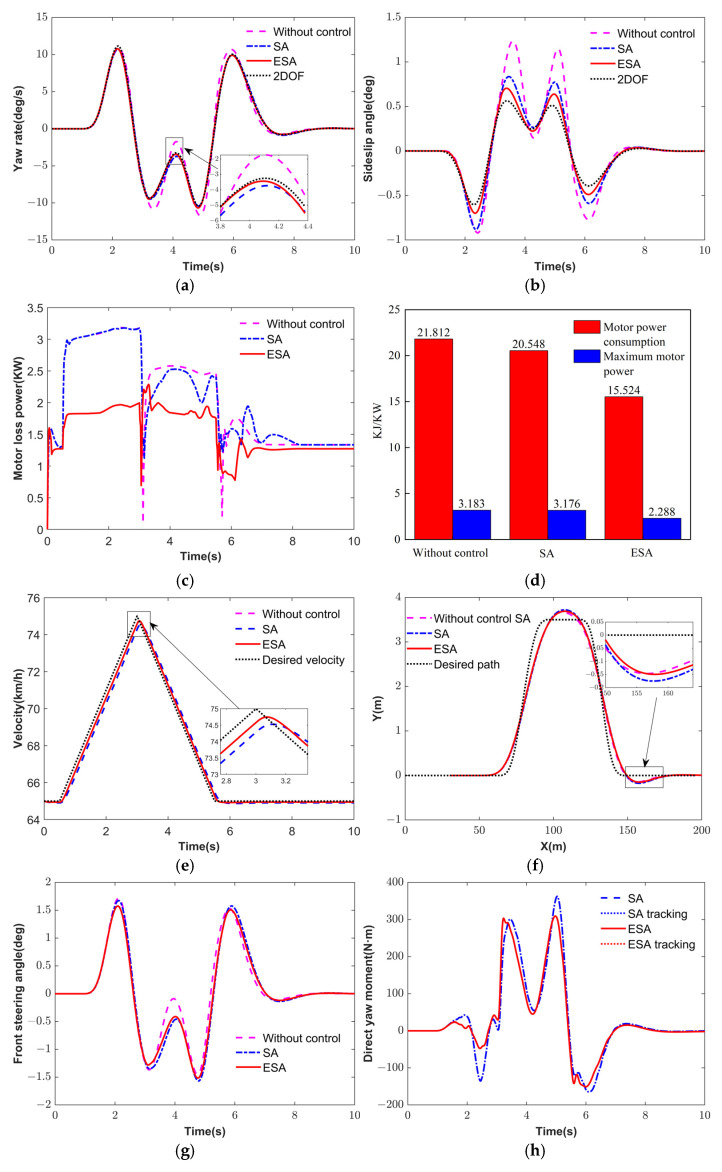

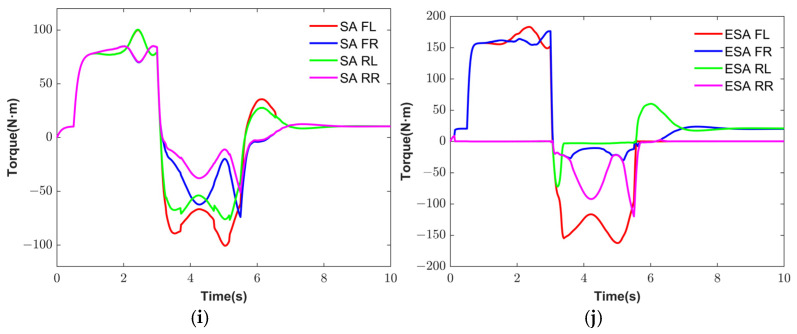
DLC simulation results. (**a**) Yaw rate. (**b**) Sideslip angle. (**c**) Motor loses power. (**d**) Velocity. (**e**) Path tracking. (**f**) Economic performance indices. (**g**) Front steering angle. (**h**) Direct yaw moment. (**i**) SA torque. (**j**) ESA torque.

Figure 16 illustrates the simulation results under the DLC. Figure 16a,b shows the simulation results of yaw rate and sideslip angle. It can be observed that both control strategies can effectively track the reference 2DOF model, thus enhancing the vehicle’s handling stability. Compared with the SA strategy, the ESA strategy reduces the RMSE of the yaw rate and the sideslip angle by 50.11% and 44.88% in Table 5, respectively. This demonstrates that the more accurate estimation of tire cornering stiffness in the adaptive MPC contributes to improved control performance.

Figure 16c,d presents the speed tracking and path tracking results, indicating that SA and ESA can effectively follow the target speed and desired trajectory. Figure 16e depicts motor loss power, and ESA has the lowest motor power consumption. As shown in Figure 16f, compared to the SA strategy, the ESA strategy reduces the peak motor loss power and consumption by 27.96% and 24.45%, respectively, confirming the superior energy efficiency of the ESA strategy that considers stability and economy. Figure 16g–j illustrates the responses of the front wheel steering angle, direct yaw moment, and driving torques. ESA strategy yields higher driving torques, improving the motor efficiency and energy conservation.

### 4.3. Statistical Validations

**Figure 17:**
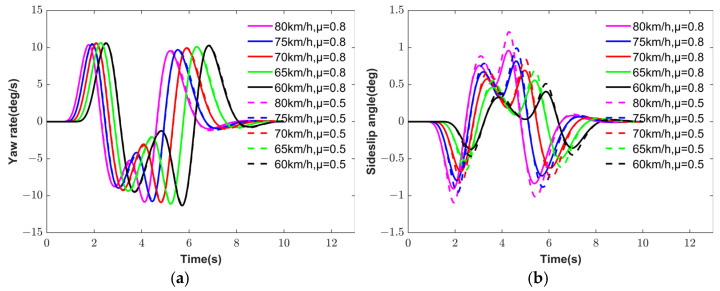
DLC simulation results. (**a**) Yaw rate. (**b**) Sideslip angle.

To verify the continuity and consistency of the hierarchical torque vectoring control strategy proposed in this paper under various simulation scenarios, ten groups of HIL simulation tests were conducted under DLC conditions, covering different vehicle speeds and TRFC. The simulation results are presented in Figure 17, which illustrates the yaw rate and sideslip angle responses under ten combinations of vehicle speeds and TRFC. As shown in Table 6, the vehicle demonstrates improved handling stability and economy in low-speed, high-TRFC scenarios.

To quantify the variability and reliability of the simulation results, the mean and 95% confidence interval of the RMSE of the sideslip angle were calculated based on Table 6. For the proposed ESA control strategy, the RMSE mean and 95% confidence interval were 0.0729 and [0.054868, 0.090968], respectively. In contrast, the EA control strategy yielded an RMSE mean of 0.1307 with a 95% confidence interval of [0.091051, 0.170394]. Compared with the EA strategy, the ESA strategy reduced the RMSE mean of the sideslip angle by 44.22% and exhibited a narrower confidence interval, indicating better stability. Similar conclusions can be drawn for the yaw rate and motor power consumption.

To further validate the statistical significance of the performance differences between the two control strategies, two-sample *t*-tests were conducted on the stability and energy consumption metrics. The *p* values for the RMSE of sideslip angle, the RMSE of yaw rate, and motor power consumption were all 0.0077, 0.0006, and 0.0011, which are less than 0.01. This indicates that the proposed ESA control strategy demonstrates a statistically significant advantage over the EA strategy. Therefore, the hierarchical torque vectoring control strategy proposed in this paper achieves superior handling stability and economy across a variety of driving conditions.

### 4.4. Real Operating Conditions Verification and Implementation Considerations

Hierarchical Torque Vectoring Control Strategy considering stability and economy for DDEVs presented in this paper has been verified on the HIL platform in terms of real-time performance and feasibility. However, it has not yet been implemented and tested in real operating conditions, which constitutes a limitation of this study. To further enhance the practicality and engineering value of the control strategy, it is necessary to thoroughly discuss the potential effects of real operating conditions on its performance.

Firstly, under actual driving conditions, more complex road scenarios may pose additional challenges. For example, under poor visibility, weak lighting, or sensor occlusion, the accuracy and stability of TRFC estimation may diminish, subsequently affecting control output. Furthermore, the vehicle’s model, such as tire characteristics and motor efficiency maps, was calibrated under laboratory conditions. These models may deviate due to loading conditions or road friction coefficient fluctuations in real operating conditions, thereby degrading control performance.

Additionally, during HIL testing, motor power consumption was primarily evaluated through the simplified Equation (30) based on motor torque, speed, and efficiency. However, energy consumption is influenced by numerous factors under actual driving conditions, such as energy recovery mechanisms, battery thermal management strategies, and communication latency, which adds complexity to the energy consumption measurement and makes the evaluation of economy more challenging.

Then, the control performance depends not only on the controller itself but also on the specific implementation scenarios and modeling assumptions.

(1) The proposed control strategy has demonstrated reductions of 23.83% and 24.45%, respectively, in motor power consumption under the FTP driving cycle and DLC conditions. Nonetheless, these results primarily reflect normal driving scenarios. The vehicle stability and economy may diminish in low friction coefficient conditions (such as snow or cat ice roads). (2) Several simplifying assumptions were made in the HIL test. For example, the tire model was represented by a standard Magic Formula, assuming corner stiffness remained constant regardless of tire pressure or wear. The motor efficiency map was treated as static, ignoring the dynamic effects of temperature and current fluctuations. These assumptions should be taken into account under actual operating scenarios. (3) If the TRFC estimation error is large due to factors such as lighting, the controller may become overly conservative, thereby affecting the ability to minimize energy consumption. Additionally, the control strategy assumes that actuator dynamics are negligible. When delays or lag effects are present in practice, these factors may diminish the performance.

In conclusion, although the proposed hierarchical torque vectoring control strategy has demonstrated its theoretical effectiveness in simulation, future work should consider its application under real-world conditions, particularly across a wider range of driving scenarios (such as snow or cat ice roads). Additionally, a more comprehensive consideration of modeling assumptions is required to further enhance vehicle handling stability and energy conservation in practice.

## 5. Conclusions

This paper proposes a hierarchical torque vectoring control strategy for DDEVs. The implementation process is as follows: Firstly, a fusion estimator based on vision and dynamics is employed to estimate the TRFC. Then, the tire model updates the tire cornering stiffness based on the estimated TRFC and the motion states of the vehicle. The adaptive MPC is used to determine the direct yaw moment. Finally, the weights of stability and energy are obtained based on the stability boundary determined by the TRFC, and the driving torques of the four wheels are optimized via the torque vectoring controller. Compared with conventional torque vectoring control strategies, the proposed approach comprehensively considers both vehicle handling stability and energy conservation. The main conclusions and contributions are as follows:

1. Compared with the traditional dynamic estimation algorithms, the TRFC fusion estimator based on vision and dynamics offers faster convergence, better robustness, and higher estimation accuracy.
2. The proposed adaptive MPC strategy updates tire cornering stiffness in real-time based on TRFC and vehicle states, significantly improving the control performance of the handling stability. The HIL simulation results under DLC conditions demonstrate that, compared with traditional DYC, the proposed strategy reduces the RMSE of yaw rate and sideslip angle by 50.11% and 44.88%, respectively, indicating enhanced vehicle handling stability.
3. An extension phase plane method is utilized to coordinate the stability and economy in the torque vectoring controller. Compared with traditional phase plane methods, the extension phase plane provides more accurate stability state assessment. Under FTP and DLC conditions, the proposed strategy reduces motor energy consumption by 3.6 KJ and 5.024 KJ, respectively, demonstrating superior energy conservation.

For future work, under extreme driving conditions, motor braking alone may be insufficient to ensure vehicle driving stability. Therefore, coordinated control strategies combining mechanical braking and motor torque vectoring should be considered. Additionally, real-vehicle experiments are necessary to further validate the effectiveness of the proposed hierarchical torque vectoring controller.

## Figures and Tables

**Table 1:** TRFC ranges for different road types.

Road Type	TRFC Range
Flagging	0.45–0.7
Asphalt	0.45–0.75
Concrete	0.45–0.75
Wet flagging	0.4–0.6
Wet asphalt	0.35–0.65
Wet concrete	0.4–0.65
Snow	0.2–0.3
Cat ice	0.05–0.2

**Table 2:** Hardware equipment details table.

Hardware Device Name	Number	Model	Manufacturer Info
64-layer LiDAR	1	Velodyne HDL-64E-S3	Velodyne Lidar, Inc., San Jose, CA, USA
Stereo camera	1	Zed 2i	Stereolabs Inc., San Francisco, CA, USA
Inertial measurement unit(IMU)	1	3DM-GX5-AHRS	MicroStrain, a subsidiary of HBK, Williston, VT, USA
RTK real-time kinematic positioning system	1	VBOX RTK	Racelogic Ltd., Buckingham, Buckinghamshire, UK
Steering angle sensor	1	Novotechnik RSC-3200	Novotechnik U.S. Inc., Southborough, MA, USA

**Table 3:** Boundary coefficients of phase plane.

TRFC	0.2	0.3	0.4	0.5	0.6	0.7
$B_{1}$	0.588	0.453	0.382	0.405	0.416	0.306
$B_{2}$	0.054	0.073	0.071	0.079	0.100	0.100

**Table 4:** Vehicle control model parameters.

Symbol	Definition	Value
m	Vehicle mass	833 kg
$I_{z}$	Vehicle yaw moment of inertia	750 kg·m^2^
$a\left(b\right)$	Distance of front (rear) axle from CG	1.1 (1.25) m
d	Track width of the vehicle	1.415 m
$N_{p}$	Predictive horizon	5
$N_{c}$	Control horizon	3

**Table 5:** Handling stability simulation results under the DLC.

Controller Error	Yaw Rate (deg/s)		Sideslip Angle (deg)		Velocity (km/h)	
	Max	RMS	Max	RMS	Max	RMS
Without control	3.3319	0.9887	0.7962	0.2625	0.7523	0.4164
SA	1.3921	0.3973	0.2569	0.0987	0.7522	0.4167
ESA	0.6302	0.1982	0.1452	0.0544	0.4386	0.2481

**Table 6:** Handling stability simulation results of the statistical validations.

Performance Index		Yaw Rate (deg/s)		Sideslip Angle (deg)		Motor Power Consumption (kw)
		Max	RMS	Max	RMS	
80 km/h, μ = 0.7	SA	1.6453	0.5839	0.2286	0.0805	22.8967
	ESA	0.5409	0.2001	0.1625	0.0593	15.9953
75 km/h, μ = 0.7	SA	1.5429	0.5632	0.1908	0.0683	19.1478
	ESA	0.4801	0.1833	0.1476	0.0555	16.0316
70 km/h, μ = 0.7	SA	1.0612	0.3649	0.2737	0.1040	17.7485
	ESA	0.4437	0.1726	0.1403	0.0533	13.8251
65 km/h, μ = 0.7	SA	1.0765	0.3709	0.2421	0.0922	16.3194
	ESA	0.4107	0.1638	0.1283	0.0485	11.8312
60 km/h, μ = 0.7	SA	1.1661	0.4042	0.2073	0.0788	14.9412
	ESA	0.4142	0.1643	0.1104	0.0412	10.4724
80 km/h, μ = 0.5	SA	1.9841	0.6351	0.6693	0.2265	22.9918
	ESA	1.4962	0.5232	0.3326	0.1137	16.0519
75 km/h, μ = 0.5	SA	1.7147	0.5572	0.5671	0.1993	19.1505
	ESA	1.1388	0.4383	0.2764	0.1013	16.2005
70 km/h, μ = 0.5	SA	1.5422	0.4941	0.4938	0.1754	17.8781
	ESA	0.9231	0.3758	0.2660	0.0971	14.0302
65 km/h, μ = 0.5	SA	1.4894	0.4720	0.4329	0.1529	16.4438
	ESA	0.8213	0.3262	0.2384	0.0869	12.0971
60 km/h, μ = 0.5	SA	1.5106	0.4792	0.3722	0.1293	15.0389
	ESA	0.7183	0.2781	0.1997	0.0724	10.5662

## Data Availability

Data Availability Statement: Data available upon request because of the project’s policies.
